# The Clinical Role of HPV Testing in Primary and Secondary Cervical Cancer Screening

**DOI:** 10.1155/2013/610373

**Published:** 2013-07-01

**Authors:** G. Hoste, K. Vossaert, W. A. J. Poppe

**Affiliations:** Department of Obstetrics and Gynaecology, University Hospitals Leuven (Campus Gasthuisberg), Herestraat 49, 3000 Leuven, Belgium

## Abstract

Traditional population-based cervical screening programs, based on cytology, have successfully reduced the burden of cervical cancer. Nevertheless limitations remain and new screening methods are emerging. Despite vaccination against the 2 most oncogenic types (HPV 16/18), cervical cancer screening will have to continue as an essential public health strategy. As the acquisition of an HR-HPV infection is critical in the progression to (pre-)cancerous cervical lesions, recent research has focused on HR-HPV detection. The sensitivity of HPV testing in primary and secondary prevention outweighs that of cytology, at the cost of slightly lower specificity. Although most of the HR-HPV infections are cleared after conization, new evidence from numerous studies encourages the implementation of HR-HPV testing and genotyping to improve posttreatment surveillance. An HR-HPV test 6 months after conization is a promising useful clinical marker to detect persistence and prevent progression. This review highlights the clinical role of HPV testing in primary and secondary cervical cancer screening.

## 1. Burden of Cervical Cancer

Cervical cancer (CC) is the third most common cancer among women worldwide (15%) and the second most common in developing countries [1]. It is estimated by the World Health Organization that every year approximately 530000 women are diagnosed with CC worldwide and 275000 women die from the disease [2].

**Figure 1:**
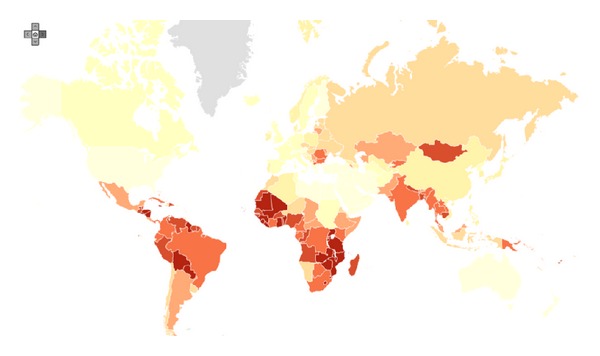
Estimated cervical cancer incidence worldwide in 2008. GLOBOCAN 2008, International Agency for Research on Cancer. The red and dark highlighted areas have the highest incidence rates.

More than 80% of the global burden occurs in developing countries, where it accounts for 13% of all female cancers. In western countries, the incidence and mortality of CC have declined substantially over the past decades, whereas in developing countries there is a slight increase in mortality (Figure 1). This is probably due to the lack of screening and the greater impact of infectious cofactors in the latter regions [3]. Age-adjusted incidence rates vary from about 10 per 100000 per year in many industrialized countries to more than 40100000 in some developing countries. More than 88% of deaths occur in low-income countries and it is predicted to increase to 91.5% by 2030 [4].

Infection with a high-risk HPV (HR-HPV) genotype has been identified as the most important etiologic risk factor for the development of CC and is the necessary step in carcinogenesis. The median age of diagnosis is 45 years, and there are two major histological types; 85% of all cases are squamous cell carcinomas (SCC) and 15% are adenocarcinomas (including adenosquamous cancers) [3]. SCC is declining whereas adenocarcinoma is increasing mainly in young women [3]. This can be explained by the hypothesis that screening methods (cytology) may be less effective in detecting adenocarcinoma.

Cervical cancer screening by regular pap smear cytology exams and/or HPV testing should, in short term, increase the likelihood of diagnosis of CC but should in the longer term decrease the likelihood of diagnosis.

## 2. Role of HPV in the Development of Cervical Cancer

HPV infection is the most common sexual transmitted disease with more than 80% of the population infected at some time in their life. In rare cases (1%), this infection will eventually lead to CC. Simultaneous infections with multiple HPV types are common [3].

Only 40 of the 200 known HPV genotypes present tropism for the anogenital mucosa and 18 of those 40 types are directly related to CC [5, 6]. Fifteen HPV types have been defined as high-risk types with strong oncogenic potential (16-18-31-33-35-39-45-51-52-56-58-59-68-73-82). These HR-HPV types account for 95% of all CC. Three additional HPV types have been identified as probable high-risk types, and 13 low-risk HPV types have been recognized.

HPV exposure is critically dependent on risky sexual behavior, such as the age of first sexual intercourse, the selection of contraceptive methods, and most importantly the lifetime number of sexual partners [1]. Most infections are transient and become undetectable within 1 to 2 years. Persistent infection is the most important risk factor for initiating malignant transformation in the cervical epithelium [6].

HR-HPV infection is an essential factor in the development of CIN and CC. When HPV acquisition is followed by HPV persistence instead of clearance, there is a high chance for progression to precancerous lesions and ultimately invasive lesions.

HPV 16 and 18 are the most common HR-HPV types worldwide and account for about 70% of all SCC and for up to 85% of all adenocarcinomas. HPV 16 is the most carcinogenic HPV genotype and HPV 18 causes a greater proportion of glandular cancers than squamous cell carcinoma. After HPV 16 and 18, the six most prevalent types that account for an additional 20% are types 31, 33, 35, 45, 52, and 58 [7].

HPV is a double stranded closed circular DNA virus with the capacity to incorporate in the human DNA. HPV 16, 18, and 45 are predominant HPV types in CC as they are more likely to integrate into the human genome than other HPV types. When CC is caused by one of these three types, CC patients are on average diagnosed 4 to 5 years earlier (44 versus 49 years) than those caused by other high-risk types [7].

**Figure 2:**
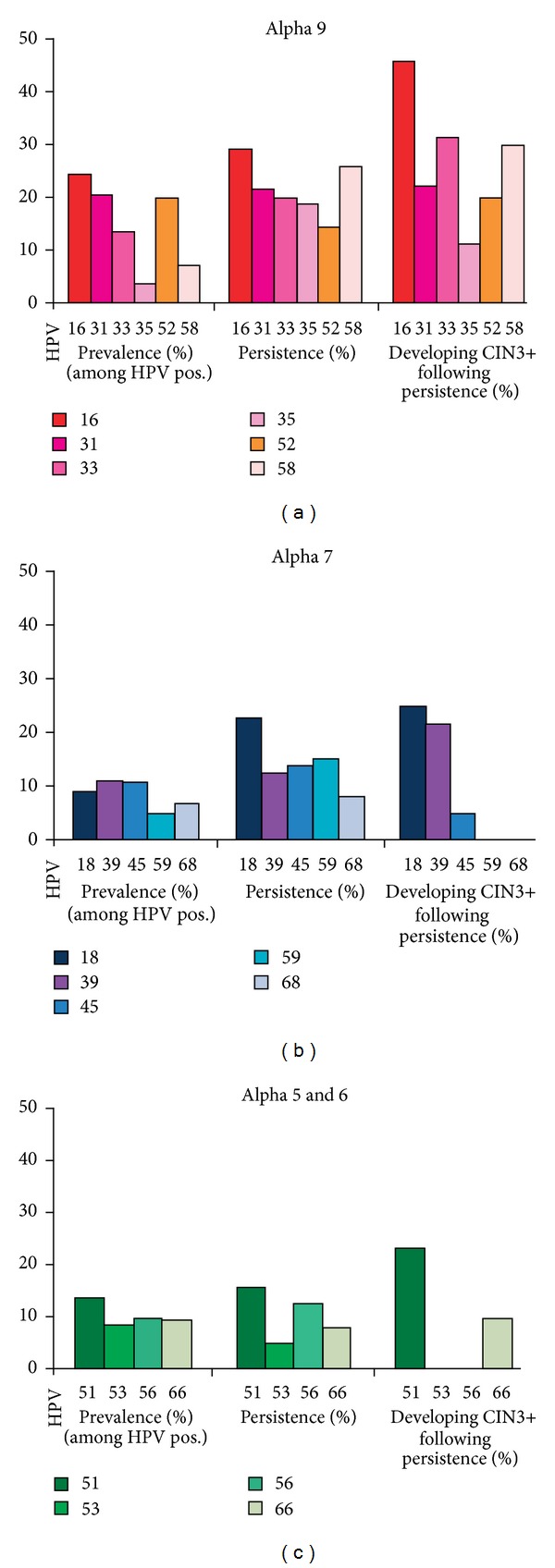
High-risk human papilloma virus type-specific prevalence of infection, percentage of women with a persistent infection and percentage of women with persistent infection who developed CIN3 or worse during follow-up period of more than 13 years after one positive test for high-risk HPV or a persistent infection (defined as two positive tests) with various specific high-risk HPV types in women with normal cytological findings [8].

HPV 16 and 18 positive LSIL are more likely to progress to CC than LSI L containing other HPV genotypes. HPV 16 and 18 account for 35% of LSIL but nearly 70% of CC worldwide. HPV 16 is more persistent and more likely to progress to CIN3+ (CIN3, carcinoma *in situ* and invasive CC) than other high risk HPV types (Figure 2) [8]. HPV-negative cervical cancer is extremely rare and is probably an artifact attributable to limitations of current detection methods or the result of loss of HPV DNA during the evolution of the tumor.

Universal vaccination against HPV 16 and 18 might prevent up to 80% of invasive CC worldwide, considering an additional cross protection against HPV strains not included in the HPV 16-18 vaccine [5, 9]. HPV vaccines are considered safe, highly efficacious, and cost-effective. Publicly funded, school-based vaccination programs that guarantee high coverage of preadolescent and young women are being introduced nowadays in many countries.

## 3. Importance of Screening

The fundamental goal of cervical cancer screening is to prevent morbidity and mortality from CC. The optimal strategy should efficiently and accurately identify those cancer precursor lesions likely to progress to invasive cancer and avoid the detection and unnecessary treatment of transient HPV infection and its associated benign lesions. Most episodes of HPV infection and many CIN1 and CIN2 cases are transient and will not develop into CIN3 or invasive CC. The potential harms associated with detecting these transient lesions include psychological distress, physical discomfort from additional diagnostic and treatment procedures, and increased risk of pregnancy complications such as preterm delivery after treatment [10]. 

New primary cervical screening guidelines have recently been introduced [11]. So far, cytology screening alone at 2- to 3-year intervals was consistently included in screening guidelines and was generally accepted as the standard of care. High-quality screening with cytology alone has indeed been very successful and has markedly reduced mortality from SCC in countries with accessible good-quality screening. This reduction is due to an increase in detection of invasive cancer at early stages and the detection and treatment of preinvasive lesions which reduces the overall incidence of invasive cancer. A Swedish nationwide population-based cohort study by Andrae et al. showed a 95% five-year relative survival ratio for women with screen detected cancers (95% CI 92–97%), whereas for women with symptomatic cancers the five-year relative survival ratio was only 69% (95% CI 65–73%) [12].

Yet false-positive cytology results were common and an increased understanding of the association between HPV and CC has led to the development of molecular HPV tests with higher sensitivity and slightly lower specificity compared with cytology. HPV tests may better predict which women will develop CIN3 or invasive cervical cancer over the next 5 to 15 years than cytology. On the other hand, as cervical carcinogenesis takes decades rather than years to occur, the relative benefits of achieving maximum sensitivity in combination with poor specificity also lead to potential harms [11, 13].

Therefore, the incorporation of HPV testing into CC screening strategies can allow both increased disease detection (improving benefits) and increased length of screening intervals (decreasing harms) [11]. Cotesting (HPV testing and cervical cytology) may result in earlier identification of women at high risk of cervical cancer. When both are negative, 5-year screening intervals are considered safe. New recommendations include age-specific screening (Table 1) [10].

CC screening should, regardless of the age of sexual debut or other risk factors, begin at the age of 21 years. In the general population under the age of 30 years, there is a low prevalence of underlying high-grade lesions and a high prevalence of transient HPV infections. Hence, the use of HPV testing as a screening tool limits the effectiveness of primary screening and could lead to unnecessary evaluation and overtreatment.

Women aged older than 65 years with adequate negative screening within the last 10 years and with no history of CIN2+ within the last 20 years should not be screened any longer. Women following a hysterectomy with removal of the cervix who have no history of CIN2+ should not be screened for vaginal cancer. Once screening is discontinued, it should not be resumed for any reason [10]. 

Screening practices should not change on the basis of HPV vaccination status. After HPV vaccination, serial screening remains necessary to further decrease cervical cancer incidence and mortality from other high-risk HPV not covered by vaccine. 

One important reason to continue screening involves inclusion of only HPV 16/18 in the first generation vaccines. Secondly, as recommendations include HPV vaccination for women up to age of 26 years, efficacy declines due to a high probability of postexposure HPV vaccination.

Even in countries with high HPV vaccination coverage, modifications to cervical screening practices are not immediately anticipated as it will take more than a decade to see the full impact of vaccination on screening outcomes. A key question is the duration of protection from HPV vaccination and the impact on age-specific cancer risks. More evidence is needed on the effect of vaccination on the HPV genotype distribution, the impact on the screening test performances, and screening adherence.

It is important to acknowledge that preventing all CC is unrealistic. No screening test has perfect sensitivity and therefore there will always be a residual cancer risk, especially for rapidly progressive CC. The optimal balance of benefit and harm should be chosen and remains a matter of discussion [11].

Technological improvements in screening are unlikely to have a substantial impact on the burden of CC incidence and mortality if they do not reach women living in low-resource, medically underserved regions. The largest immediate gain in reducing the burden could be attained by increasing access to screening among women who are currently unscreened or screened infrequently. The incorporation of HPV testing may be advantageous as it provides longer term safety following a negative test. This is a useful characteristic for women who are screened infrequently.

## 4. HPV Testing for Screening

In a meta-analysis in 2006, HPV testing has been shown to have greater sensitivity (+37%) but lower specificity (−7%) for CIN2+ and better reproducibility than cytology using a positive cut-point of LSIL [14].

This screening tool should not be used in women younger than 30 years because of the high prevalence of HPV in young adult women. Therefore, for women aged 21 to 29 years, screening with cytology alone every 3 years is recommended as it provides the best balance of benefits and harms of screening in this age group.

New recommendations state that women aged 30 to 65 years should be screened with cytology and HPV testing every 5 years or with cytology alone every 3 years. Based on risks and harms assessment, cotesting is preferred to cytology alone. The addition of HPV testing results in an increased detection of CIN3 with a concomitant decrease in CIN3+ detected in subsequent rounds of screening. This increase in diagnostic lead time translates into lower risk following a negative screen, permitting a lengthening of screening intervals with similar or lower incident cancer rates than screening with cytology alone at shorter intervals. The introduction of HPV testing also enhances the identification of women with adenocarcinoma and its precursors.

The main harms associated with adding HPV testing can be alleviated by extending the screening interval to 5 years, thereby reducing the detection of transient HPV infections and related lesions. Cotesting more frequently than recommended is predicted to exacerbate the harms by increasing the number of colposcopic referrals and treatments.

Women cotesting HPV positive, cytology negative should not be referred directly to colposcopy. Cotesting should be repeated after 12 months or immediate HPV 16/18 genotype specific testing should be performed. Only those women testing positive on either test (HPV positive or LSI L or more severe lesions) or testing positive for HPV 16/18 should be referred to colposcopy.

The risk of precancerous lesions following HPV-negative ASC-US cytology is very low and not qualitatively different from a negative cotest. Because of the very low CC risk observed in these cases, continued routine screening is recommended. Women with HPV-positive ASC-US or more severe cytology should be referred to colposcopy, regardless of their HPV status.

HPV testing alone for primary screening appears promising in women aged 30 years and older. A negative HPV test provides greater reassurance against CI N3+ in the subsequent 5 to 7 years than cytology alone and is nearly as reassuring as a negative cotest [10]. Therefore, an acceptable screening interval should be comparable to that of cotesting. However, the data of HPV testing alone are limited by a lack of long-term followup. Further research is needed to support HPV testing alone for screening. 

## 5. HPV Testing in Followup after Treatment

Women with high-grade cervical lesions are treated by local excision or ablation to prevent progression to invasive CC. Although conization is proven to be an effective treatment in removing CIN, it does not necessarily mean eradication of the virus. Failure of treatment for CIN3 has been reported to vary between 5 and 25% [15]. In a cure setting, the main focus is the early detection of any residual HPV infection after treatment whereas a high sensitivity is essential. Awareness of the role of persistent HR-HPV infection has raised the willingness to implement HPV testing to detect these high risk populations.

**Figure 3:**
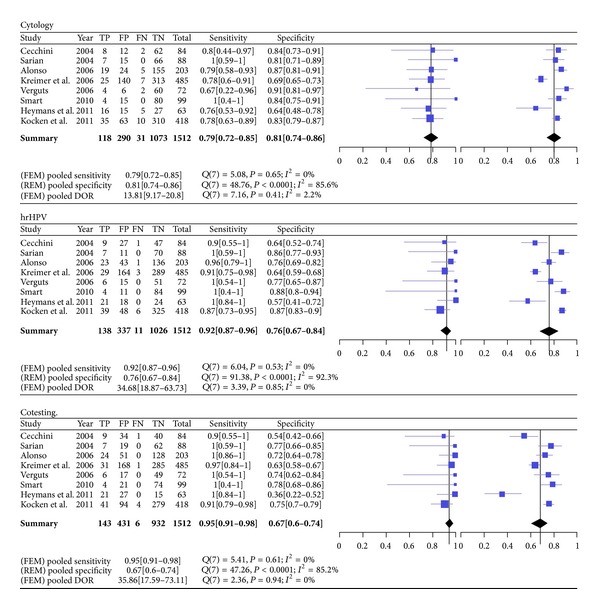
Meta-analysis of the sensitivity and specificity, including pooled estimates of testing 6 months after treatment with cytology, HR-HPV, or cotesting. Legend: forest plots of sensitivity (left) and specificity (right). TP = true positives, FP = false positives, FN = false negatives, TN = true negatives, FEM = fixed effect model, REM = random effects model, DOR = diagnostic odds ratio [16–21].

**Figure 4:**
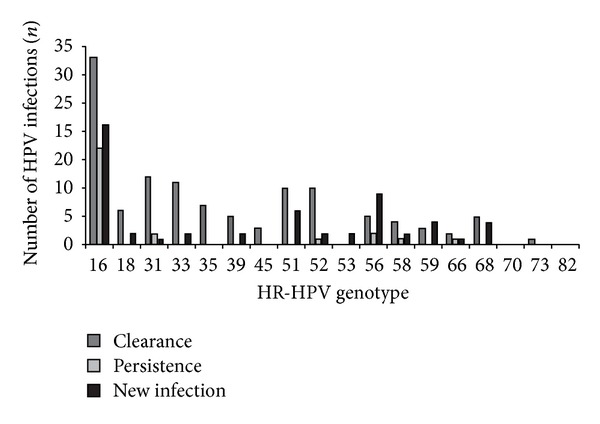
Ninety-eight women treated by LLETZ for CIN2+ had liquid base cytology samples taken before and after treatment for HPV testing. The number of HR-HPV infections that were cleared are indicated in dark grey, the infections that persisted after treatment are indicated in light grey, and all new infections are shown in black [22].

In a recent meta-analysis, Kocken et al. described the value of testing for cytology and/or HR-HPV in the surveillance of women treated by conization for CIN2+ [16]. At the 6-month posttreatment examination, HR-HPV testing has a significantly higher sensitivity than cytology, indicated by a relative sensitivity of 1.15 (95% CI 1.06–1.25), without decreasing the specificity (relative specificity 0.95, 0.88–1.02). Combined analysis of cytology and HR-HPV test predicted high-grade disease recurrence with higher sensitivity than the separate individual tests (Figures 3 and 4) [16–22].

This review confirms the advantage of implementing HR-HPV testing as virological surveillance in predicting therapeutic failure [9, 11]. As even the sensitivity of HR-HPV testing (or cotesting) is not sufficiently high to rely on a single test moment, repeat testing is necessary to identify all women at risk for residual disease.

Evidence-based posttreatment followup consists of retesting at 24 months for women with a negative cotest at six months after treatment [15, 23].

Moreover, other recent studies firmly stress the importance of type-specific persistence after treatment. A retrospective case-control study by McCredie et al. demonstrated that a type-specific persistent HPV infection at first control 6 months after conization was an independent risk factor associated with a higher frequency of recurrent CIN in the next 24 months [13]. Compared to HR-HPV testing, HPV genotyping predicts residual disease with the same sensitivity and with a significantly higher specificity (ratio: 1.43, CI: 1.28). According to the HPV genotype, different risk levels for progression could be defined. HR-HPV infections caused by HPV 16 or HPV 18 are more frequently associated with persistence than other HR types and should be monitored more intensively [24]. In this study of Heymans et al. HPV 16 infection cleared significantly less in women with recurrent high-grade CIN compared with women without recurrence. This result is in line with other recent findings that HPV 16 exhibits a lower clearance rate than other high risk HPV types [25, 26].

In the future, the introduction of HPV genotyping promises the potential to refine the algorithm for the management of HR-HPV positive women after treatment. However, further work is required to investigate the role of HPV genotyping, cofactors, viral load determination, and molecular markers to recommend the most appropriate test for patient management after treatment.

## Figures and Tables

**Table 1:** Summary of recommendations that reflect the best evidence-based practice for the prevention of CC morbidity and mortality through currently available screening tests that maximize protection against CC while minimizing the potential harms associated with false-positive results and overtreatment.

Recommended screening method^a^	Management of screen results	Comments
No screening		HPV testing should not be used for screening or management of ASC-US in this age group

Cytology alone every 3 y	HPV-positive ASC-US^b^ or cytology of LSIL or more severe: refer to ASCCP guidelines	HPV testing should not be used for screening in this age group
	Cytology negative or HPV-negative ASC-US^b^: rescreen with cytology in 3 y	

	HPV-positive ASC-US or cytology of LSIL or more severe: refer to ASCCP guidelines^2^	
HPV and cytology “cotesting” every 5 y (preferred)	HPV positive, cytology negative: Option 1: 12-mo followup with cotesting Option 2: Test for HPV 16 or HPV 16/18 genotypes (i) if HPV 16 or HPV 16/18 positive: refer to colposcopy (ii) if HPV 16 or HPV 16/18 negative: 12-mo followup with cotesting	Screening by HPV testing alone is not recommended for most clinical settings
	Cotest negative or HPV-negative ASC-US: rescreen with cotesting in 5 y	

Cytology alone every 3 y (acceptable)	HPV-positive ASC-US^b^ or cytology of LSIL or more severe: refer to ASCCP guidelines^2^	
	Cytology negative or HPV-negative ASC-US^b^: rescreen with cytology in 3 y	

No screening following adequate negative prior screening		Women with a history of CIN2 or a more severe diagnosis should continue routine screening for at least 20 y
No screening		Applies to women without a cervix and without a history of CIN2 or a more severe diagnosis in the past 20 y or cervical cancer ever

Follow age-specific recommendations (same as unvaccinated women)		

^a^Women should not be screened annually at any age by any method. ^b^ASC-US cytology with secondary HPV testing for management decisions [10].
